# Differential Survivability of Two Genetically Similar *Salmonella* Thompson Strains on Pre-harvest Sweet Basil (*Ocimum basilicum*) Leaves

**DOI:** 10.3389/fmicb.2021.740983

**Published:** 2021-12-07

**Authors:** Ye Htut Zwe, Michelle Mei Zhen Ten, Xinyi Pang, Chun Hong Wong, Dan Li

**Affiliations:** Department of Food Science and Technology, National University of Singapore, Singapore, Singapore

**Keywords:** *Salmonella* Thompson, pre-harvest basil, hypersensitive response, expression polymorphism, type III secretion system

## Abstract

Although conventionally considered an animal pathogen, recent evidence increasingly suggests that fresh produce may act as significant transmission vehicles and alternative hosts to *Salmonella*. This study reports the differential survivability of two genetically similar *Salmonella* Thompson strains (ST 889B and ST 688C) on the adaxial surface of pre-harvest basil (*Ocimum basilicum*) leaves. Upon inoculation, two distinct phenomena, a dried water-print or a macroscopic lesion, were observed within 24 h. ST 889B survived better than ST 688C on healthy-looking leaves without lesions, possibly due to its higher biofilm-forming ability. Both strains survived better on the leaves with lesions than on the healthy-looking leaves (ST 688C: 4.39 ± 0.68 vs. 2.18 ± 0.29; ST 889B: 4.78 ± 0.12 vs. 2.83 ± 0.18 log CFU per sample at 6 days post-inoculation). ST 889B caused the formation of lesions at a higher frequency [70/117 leaves (59.8%)] than ST 688C [35/96 leaves (36.5%)]. Thus, we highlighted two distinct *Salmonella* survival strategies in the basil pathosystem and demonstrated gene expression polymorphism (variations in the expression of the same set of genes) as an indispensable strategy in the colonization of plants as hosts by the human pathogens.

## Introduction

*Salmonella enterica*, which causes non-typhoidal salmonellosis, is an important foodborne pathogen estimated to cause up to 11% of the 9.4 million foodborne illnesses in the United States annually. It is also one of the most virulent and fatal foodborne pathogens, causing up to 35 and 28% of all foodborne-related hospitalizations and deaths, respectively, in the United States (Scallan et al., 2011). Its impact is also felt in smaller nations like Singapore, where 68% of all reported food- and waterborne disease cases consisted of non-typhoidal salmonellosis (Zwe and Yuk, 2017). Fresh produce has been identified as an important vehicle for the transmission of *Salmonella*. Culinary herbs such as basil have drawn particular attention for their food safety since once fresh culinary herbs are contaminated, completely removing or killing pathogens is unlikely. Large-scale *S*. Senftenberg and *S*. Anatum outbreaks have been reported in recent history due to contaminated fresh basil consumption in multiple countries (Pezzoli et al., 2008; Pakalniskiene et al., 2009). Therefore, it is crucial to understand how *Salmonella* survives in the pre-harvest basil plants for its food safety implications.

In this study, we report the differential capabilities of two *S*. Thompson isolates in colonizing and surviving on the basil plants. ST 889B was isolated from basil in Belgium in 2013 (Delbeke et al., 2015), and ST 688C was a clonal subculture of the 1999 California coriander outbreak clinical-isolate RM1894 (Li and Uyttendaele, 2018). These two *S*. Thompson isolates were of near-identical genetic makeup and thus served as valuable study materials to examine the plant–pathogen interactions between *Salmonella* and fresh produce, offering insights that may be critical to improving the microbial safety of plant produce.

## Materials and Methods

### Bacterial Strain and Culture Conditions

ST 688C is a clonal subculture of the *S*. Thompson strain RM1984 clinically isolated from a salmonellosis patient during the California coriander outbreak in 1999, kindly provided by Dr. Maria Brandl (U.S. Department of Agriculture, Agricultural Research Service, Albany, CA, United States). ST 889B was previously isolated from basil (Delbeke et al., 2015), kindly provided by Prof. Mieke Uyttendaele (Ghent University). The frozen cultures of bacteria were activated by transferring twice consecutively (37°C, 24 h) in sterile tryptic soy broth (TSB, Oxoid, Basingstoke, Hampshire, England). The stock cultures of bacteria were maintained on tryptic soy broth (TSA, Oxoid) plates stored at 4°C. A single colony from TSA was transferred into 10-ml TSB and incubated at 37°C for 24 h. A 1-ml portion of the bacterial suspension was centrifuged at 9,000 × *g* at 4°C, washed twice in phosphate-buffered saline (PBS; Vivantis, Inc., Oceanside, CA, United States), serially diluted in 0.1% peptone water (PW; Oxoid) to appropriate concentration to obtain the working cultures of bacteria for downstream experiments.

### Whole Genome Sequencing and Bioinformatic Analysis

The DNA from the 24-h cultures of bacteria were extracted using the GeneJET Genomic DNA Purification Kit (Thermo Fisher Scientific, Vilnius, Lithuania) following the manufacturer’s instructions. Whole genome sequencing (WGS) was performed by NovogeneAIT Genomics Singapore Pte Ltd. using Illumina Hiseq4000, and 350 bp insert DNA library preparation.

Raw reads were assembled into contigs using Assembler 1.2 publicly available at the Center for Genomic Epidemiology by the Technical University of Denmark^1^ using default parameters. Assembled contigs were analyzed for resistance determinants, multilocus sequence type (MLST), and core genome multilocus sequence type (cgMLST) using ResFinder 4.0 (Zankari et al., 2012; ResFinder Database 2020-04-08 and PointFinder Database 2019-07-02), MLST 2.0 (Larsen et al., 2012), and cgMLSTFinder 1.1 (Alikhan et al., 2018), respectively, using default parameters. The average nucleotide identity between ST 688C and ST 889B was analyzed using the ANI Calculator (Yoon et al., 2017). The contigs were deposited to the GenBank, National Center for Biotechnology Information (NCBI)^2^ under accession numbers JAFFRV000000000 (ST 688C) and JAFDOS000000000 (ST 889B) of BioProject PRJNA697834.

Single-nucleotide polymorphism tree for phylogenetic analysis was performed using the CSI Phylogeny 1.4 (Kaas et al., 2014) using default parameters. All known genome assemblies of 46 *S*. Thompson strains were downloaded from the NCBI. Three genomes were found to be outgroups from the others in an initial trial and were subsequently excluded. A group of 43 genomes, ST 688C, and ST 889B, were used to construct a phylogenetic tree based on SNPs with *S*. Thompson RM6836 as the reference. The resulting tree was visualized using the public available FigTree 1.4.4 software.^3^ Bootstrap values displayed were automatically calculated by the proprietary algorithm of the CSI Phylogeny 1.4 software.

### *Salmonella* Survivability in Sterile Basil Juice

A 100 g portion of *Ocimum basilicum* sweet basil leaves and stems from the freshly bought basil plants were immediately harvested and added to 200 ml of deionized (DI) water and homogenized in a food processor. The solid particles in the resultant suspension were removed by first filtering through a mesh strainer, followed by multiple rounds of centrifugation (9,000 × *g*, 4°C, 10 min) until no visible solid particles were present throughout the liquid. The basil juice was sterilized by passing through 0.22 μm filters.

A 100 μl portion of the working culture of ST 688C or ST 889B (*ca*. 10^9^ CFU/ml) in 0.1% PW was inoculated into 30 ml of sterile basil juice in 50-ml falcon tubes. The samples were stored loosely capped at 25°C over 3 days. A 100 μl portion of the sample was drawn from each sample at 0 (immediately after inoculation), 1, 2, and 3 days post-inoculation (dpi), serially diluted in 0.1% PW and enumerated on xylose lysine deoxycholate (XLD; Oxoid) agar.

### Biofilm-Forming Ability Assay

As described by Lee et al. (2011), crystal violet biofilm assay was employed with slight modifications to determine the biofilm-forming ability of ST 688C and ST 889B. Briefly, working cultures of bacterial cells were serially diluted in 0.1% PW while the final dilution was performed in either 1/10 TSB (TSB:water of 1:9) or sterile basil juice to a final concentration of 10^5^ CFU/ml. For every combination of bacterial strain and medium, a 200 μl portion of the sample per well was added to six wells in a 96-well plate. After incubating at 25°C for 24 h, each well was washed three times with sterile DI water, stained with 200 μl of 0.1% (wt/vol) crystal violet (Sigma-Aldrich, St. Louis, MO, United States) solution for 20 min at room temperature, rewashed three times with DI water, and incubated with 200 μl of 95% ethanol at 4°C for 45 min to extract dye from the biofilm. Absorption at 595 nm was measured using a Multiskan FC microplate photometer (Thermo Fisher Scientific, Shanghai, China).

### Construction of GFP-Tagged Strains and Fluorescence Confocal Microscopy

The procedures for preparing competent *Salmonella* cells and the construction of GFP-tagged *Salmonella* strains through the transformation with pGFPuv plasmid have been previously described (Ma et al., 2011). Briefly, a 250 μl portion of overnight cultures of ST 889B incubated at 37°C in at 37°C Luria-Bertani (LB, Invitrogen, CA, United States) broth was transferred into 25 ml of LB broth and incubated at 37°C for 3 h in the shaker incubator shaking at 200 rpm. The resultant bacterial suspension was then cooled in ice for 10 min, centrifuged (4,000 rpm, 4°C, 10 min), resuspended in 10 ml of 0.1 M CaCl_2_, centrifuged, and resuspended again in 2 ml of 0.1 M CaCl_2_ with 15% glycerol medium. The pGFPuv plasmid was extracted using the Qiagen plasmid miniprep kit (Qiagen, Hilden, Germany), following the manufacturer’s instructions. The transformation was performed by the heat-shock method. Briefly, 5 μl of the plasmid was mixed into 50 μl of competent cells and kept on ice for 30 min before transferring to a 42°C incubator for 30 s and transferring back on ice for 5 min. A 950 μl portion of LB broth was added to the heat-shocked bacterial-plasmid mix and incubated at 37°C for 1 h while shaking at 250 rpm. The suspension was plated onto LB agar with 100 μg/ml ampicillin to recover the transformants. The GFP-tagged bacteria were grown in TSB with 100 μg/ml ampicillin before washing and appropriately diluted as previously described for inoculation. The observation of GFP-tagged bacteria was performed with fluorescence confocal microscopy Olympus Fluoview FV1000 using the emission band 505–525 nm and the autofluorescence from the leaves, using emission band 525–650 nm.

### *Salmonella* Survivability on Pre-harvest Basil (*Ocimum basilicum*) Leaves

Pre-harvest *O. basilicum* sweet basil plants (*ca*. 2 months of age) were purchased from a local supermarket. A 300-W LED lamp (Melonfarm) was used in the 32″ × 32″ × 63″ grow tent (MelonFarm, China) to supply light with a photoperiod of 18 h and a dark period of 6 h. The temperature (26 ± 2°C) and relative humidity (58 ± 8%) in the grow tent were monitored using an EL-USB-2-LCD+ logger (Lascar Electronics Ltd., United Kingdom). Newly purchased plants were subjected to a 24-h acclimatization period to the new environment in the grow tent before inoculation with bacteria.

A 100 μl portion working culture of ST 688C or ST 889B (*ca*. 10^9^ CFU/ml) in PBS was inoculated onto the adaxial side of an inverted basil leaf (to form a “cup-like” depression to assist inoculation) in a roughly 1 cm^2^ area and dried ambiently. Immediately after drying (0 dpi) and at other time points (1 and 6 dpi), leaves for each bacterial strain were harvested to be enumerated. The 1 cm^2^ portion from each leaf where the inoculum has dried was cut and mixed with 5 ml of PBS in a small vacuum-sealed stomacher pouch. The leaf’s surface was lightly massaged for 1 min, followed by a light crushing using a pestle to release any bacterial cells that may be internalized within the leaf structure. The resultant suspension was then serially diluted to the appropriate concentration in 0.1% PW and spread plated on XLD (Oxoid) agar plates and incubated at 37°C for 24 h.

### Gene Expression Assay

RNA from freshly dried bacterial inoculum on basil leaves was extracted using the RNeasy Mini Kit (Qiagen) and was reverse transcribed using the GoScript^™^ Reverse Transcriptase kit (Promega Corp., Madison, WI, United States) following the manufacturer’s instructions to obtain the cDNA. The primers used in this study were designed using the Primer-BLAST tool^4^ and are described in Table 1. The single-copy chromosomal gene *hcaT* was used as the reference gene (Barak et al., 2007). The assay was performed in 10 μl reaction volumes with 5 μl of GoTaq^™^ qPCR Master Mix (Promega), 0.25 μl each of the forward and reverse primer at 10 μmol/l concentration, and 1 μl of cDNA template. Threshold cycle (C_T_) values were measured using the StepOnePlus Real-Time PCR System (Applied Biosystems, Carlsbad, CA, United States). The thermal cycling conditions were as previously described (Yang et al., 2014). The fold change in gene expression was calculated as previously described (Pfaffl, 2001).

**Table 1:** Primers used in this study.

Function	Primers	5' to 3'	Product size
Biofilm	bcsA_F	GTCCCACATATCGTTACCGTCCTG	119
	bcsA_R	CGCCGCATCATTTCTTCTCCC	
	wcaD_F	CTGCCGCCAGTAAGGATAAT	111
	wcaD_R	CATTTCCGCGCATAACCACC	
	csgB_F1	GGTCAAGTCGGCACGGATAA	103
	csgB_R1	GGTCGACTTTCGCCCGATTA	
	bapA_F	CGGTGAATTCGTCGTTACGC	115
	bapA_R	GTCGGAAGCGGGAAAATTCG	
SPI-1 T3SS	prgH_F	GCTAAAACCTGACGAGCCCA	127
	prgH_R	CCGCAGAGCTCGATTCGTTA	
PAMP signature	fliC_F	GTTCAACGGCGTGAAAGTCC	115
	fliC_R	ACCCAGGGTCTGAGAGTTGA	
Housekeeping	hcaT_F	CCTGCAAACGAATCACCT	113
	hcaT_R	GCCGTGGCTGATTGTGATA	

*SPI-1, Salmonella pathogenicity island-1; T3SS, type III secretion system; PAMP, pathogen-associated molecular patterns*.

### Statistical Analysis

Each bacterial count data point was derived from three independent experiments with two biological replicates (*n* = 6). Biofilm-forming assay data consisted of two independent experiments with six wells each. Fold change in gene expression data was derived from two independent experiments, each consisting of RNA extracted from three biological samples which were polled together. Unpaired t-test, which can be accessed online at https://www.graphpad.com was used to compare bacterial count, biofilm-forming ability, and fold change in gene expression data set.

## Results and Discussion

### WGS Analysis

**Figure 1:**
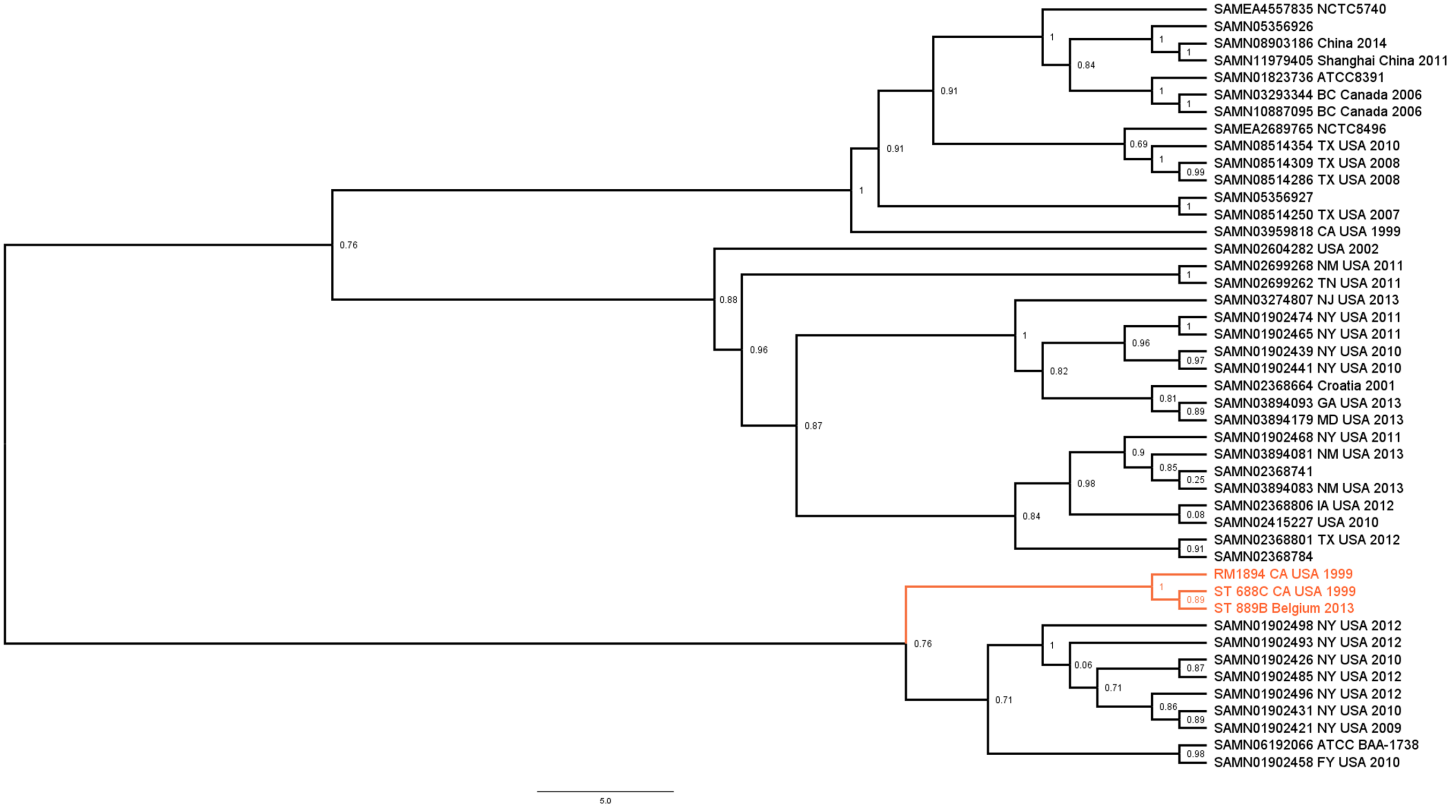
Maximum likelihood SNP tree illustrating the phylogenetic relationship between ST 688C, ST 889B, the 1999 California coriander outbreak clinical strain RM1984 (three isolates of interest highlighted in color) and all other publicly available closely related members of *S*. Thompson isolates. Genomes downloaded from NCBI were denoted with their Biosample accession numbers followed by the place and year of isolation, if available. Numbers denote bootstrap values.

Whole Genome Sequencing analysis revealed that ST 688C and ST 889B had identical MLST (Larsen et al., 2012) and cgMLST (Alikhan et al., 2018) of 26 and 5529, respectively. They also carried the identical chromosomal mutations *gyrA* S83Y and *parC* T57S. The acquired resistance gene *aac(6′)-Iaa* was present with an ID match of 97.72%, and both genomes featured identical point mutations in ten bases. The exact sequence of the *aac(6′)-Iaa* gene in the form of ResFinder 4.0 (Zankari et al., 2012) output text file from both ST 688C and ST 889B is available as Supplementary Figure S1. The two genomes shared an average nucleotide identity of 99.98% and an identical GC content of 52.23%. A SNP tree was constructed by including all known *S*. Thompson genomes from the NCBI database to investigate their phylogeny (Figure 1). ST 688C was genetically similar to the RM1894 outbreak strain and ST 889B. The SNP differences between the genomes were as follows: ST 688C and RM1894 (6 SNPs), ST 688C and ST 889B (9 SNPs), and ST 889B and RM1894 (9 SNPs). These three isolates formed a distinct clade (Figure 1; colored) whose most genetically similar clade consists of *S*. Thompson strains isolated exclusively from the United States from 2009 to 2012 except for the *S*. Thompson ATCC BAA 1738. In short, ST 688C and ST 889B are genetically similar to each other and the original RM1894 outbreak strain.

### Inoculation of *S*. Thompson Onto Pre-harvest Basil Leaves

**Figure 2:**
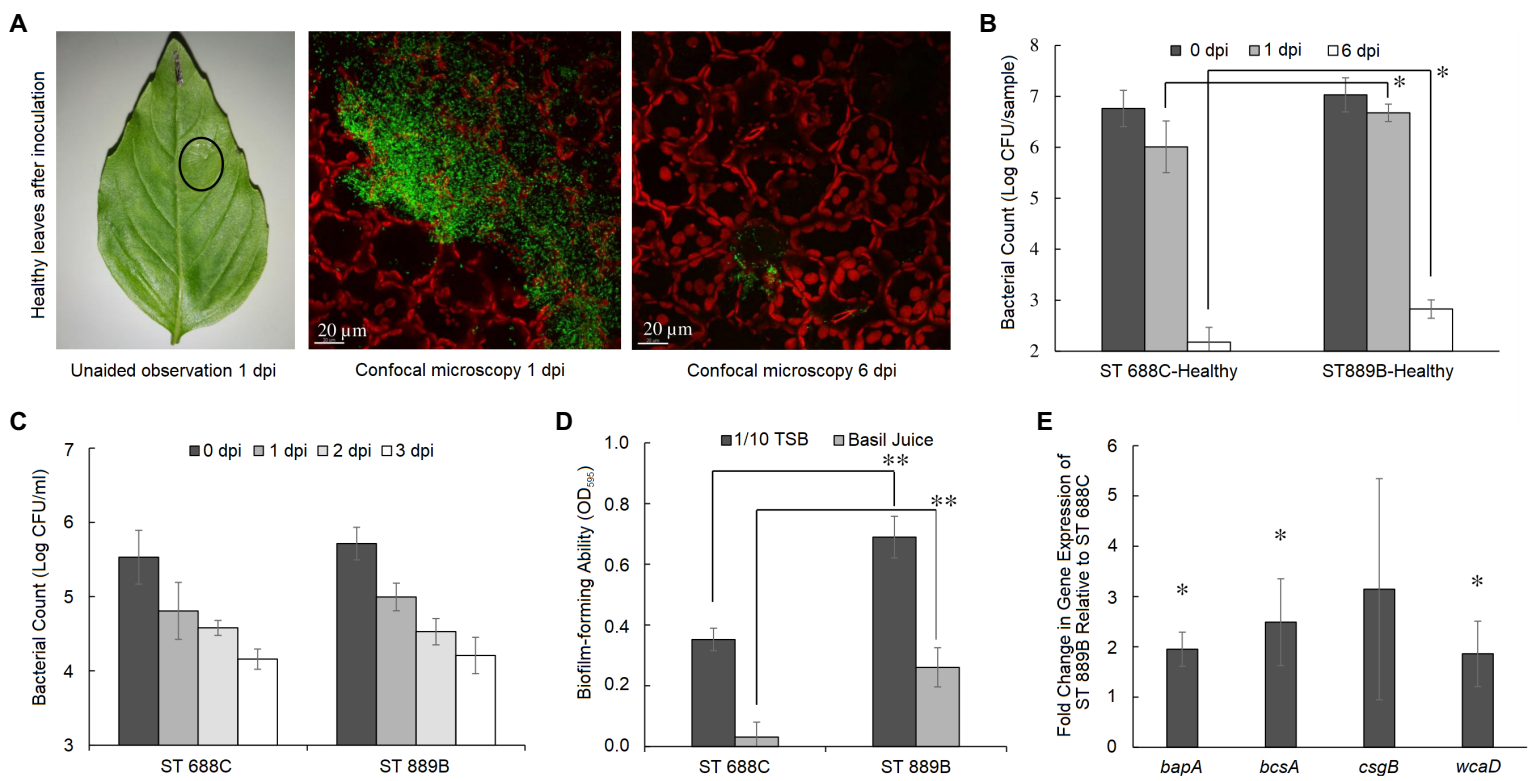
**(A)** A representative healthy-looking basil leaf as seen unaided at 1 dpi, and under confocal microscopy at 1 and 6 dpi (from left to right) where red signals represent leaf cells while green signal represents GFP-tagged *Salmonella* cells; **(B)** bacterial counts of ST 688C and ST 889B on healthy-looking leaf tissue at 0, 1, and 6 dpi; and **(C)** in sterile basil juice at 0, 1, 2, and 3 dpi; **(D)** biofilm forming ability of ST 688C and ST 889B in 1/10 TSB and sterile basil juice; **(E)** fold change in gene expression of biofilm formation genes in ST 889B relative to ST 688C. Values linked by lines and denoted by ^*^ and ^**^ represent significant difference at *p* < 0.05 and < 0.01, respectively, except in **(E)**, where ^*^ denotes significant difference (*p* < 0.05) to a theoretical mean of 1, which is the relative gene expression level of ST 688C. Bacterial counts in **(B,C)** were the average of three experiments, while that of biofilm forming ability in **(D)** and fold change in gene expression in **(E)** were the average of two experiments. Error bars denote standard deviation.

**Figure 3:**
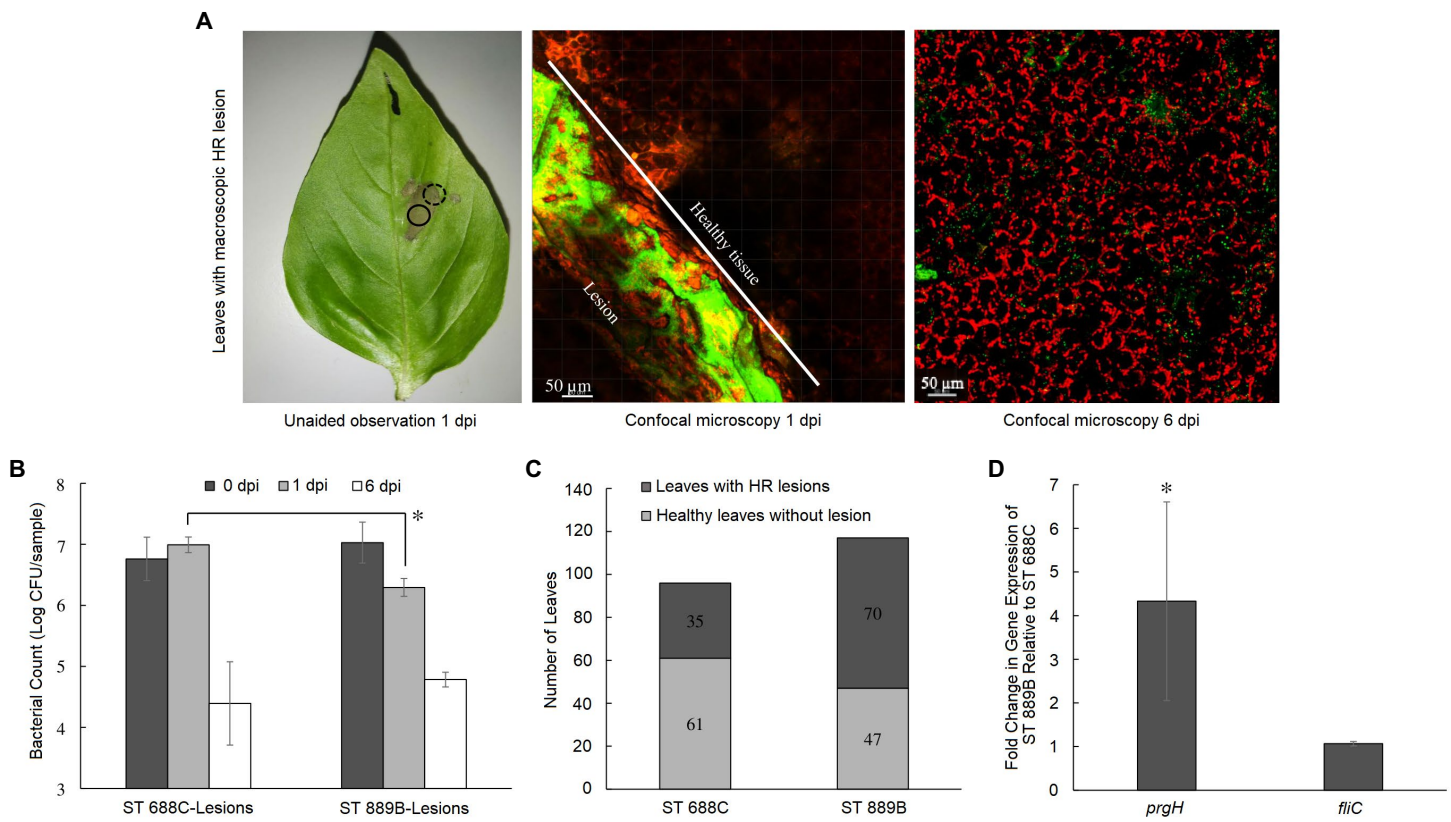
**(A)** A representative basil leaf with macroscopic HR lesion as seen unaided at 1 dpi, and under confocal microscopy at 1 (dotted circle showing the border between healthy tissue and the lesion separated by the white line) and 6 dpi (full circle showing the lesion; from left to right) where red signals represent leaf cells while green signal represents GFP-tagged *Salmonella* cells; **(B)** bacterial counts of ST 688C and ST 889B on leaf tissue with HR lesion at 0, 1, and 6 dpi; **(C)** number of resultant healthy-looking leaves without HR and unhealthy leaves with HR following inoculation with ST 688C or ST 889B; **(D)** fold change in gene expression of select HR-related genes in ST 889B relative to ST 688C. Values linked by lines and denoted by ^*^ represent significant difference at *p* < 0.05 except in **(D)**, where ^*^ denotes significant difference (*p* < 0.05) to a theoretical mean of 1, which is the relative gene expression level of ST 688C. Bacterial counts were the average of three experiments in **(B)**, while that of the fold change in gene expression in **(D)** was of two experiments. Data on the number of leaves with and without HR lesions upon inoculation with ST 688C and ST 889B **(C)** were derived across 10 and 12 different pre-harvest basil plants, respectively. Error bars denote standard deviation.

Upon inoculation of ST 688C and ST 889B on the adaxial surface of pre-harvest basil leaves, it was observed that the bacterial inoculum might either dry up on the leaf surface without eliciting any visible response from the leaf, i.e., no macroscopic lesions (referred to as healthy-looking leaf samples hereafter) as shown in Figure 2A or it may result in the formation of macroscopic lesions as shown in Figure 3A. The lesions fully developed within 24-h post-inoculation and were restricted to the leaf’s portion in contact with the inoculum. Once fully formed, no further enlargement of the lesions was observed throughout the experiment (6 days).

### Survivability of *S*. Thompson on Healthy-Looking Leaves

On the healthy-looking leaves, fluorescence confocal microscopy revealed that despite many GFP-tagged bacteria (emitting green fluorescence signal) at 1-day post-inoculation (dpi), very few remained by 6 dpi (Figure 2A). ST 889B was found to have survived significantly (*p* < 0.05) better at both 1 (6.68 ± 0.17 vs. 6.01 ± 0.51 log CFU/sample) and 6 dpi (2.83 ± 0.18 vs. 2.18 ± 0.29 log CFU/sample) than ST 688C (Figure 2B).

As basil is known to produce antimicrobial substances such as linalool (Bagamboula et al., 2004; Beier et al., 2014), we first investigated if the two *S*. Thompson isolates had differential resistance to the potential antimicrobial basil-derived substances in freshly prepared sterile basil juice. The survivability of the two *S*. Thompson strains in sterile basil juice was determined over 3 days, and no significant differences in bacterial counts between the two strains were observed (*p* > 0.05, Figure 2C). On average, the counts of both strains reduced from ~5.6 log CFU/ml initially to ~4.2 log CFU/ml by 3 dpi.

Biofilm formation can increase *Salmonella*’s resistance toward stresses such as desiccation (Gibson et al., 2006; White et al., 2006, 2010) and, consequently, its ability to persist on abiotic surfaces and non-host environments (Gibson et al., 2006; White et al., 2006, 2010; Vestby et al., 2009). There is accumulating evidence showing that *Salmonella* can adhere to and from biofilms on plant surfaces, leading to persistent survival and colonization (Yaron and Römling, 2014; Maruzani et al., 2019). Accordingly, we compared the two *S*. Thompson isolates’ biofilm-forming ability in both bacterial culture broth (1/10 tryptone soya broth, TSB; 1:9 TSB:DI water) and freshly prepared basil juice. Indeed, ST 889B showed significantly higher (*p* < 0.01) biofilm-forming ability than ST 688C in both 1/10 TSB and basil juice (Figure 2D). Accordingly, ST 889B also showed a higher mean fold change in gene expression level across the four biofilm formation genes tested in this study: *bapA* (1.95-fold), *bcsA* (2.49-fold), *csgB* (3.15-fold), and *wcaD* (1.86-fold) as compared to ST 688C after the inoculum have dried on pre-harvest basil leaves (Figure 2E).

These results suggest that in the scenario where *Salmonella* failed to elicit a visible reaction from the basil leaves upon inoculation, their survival strategy is more comparable to that of on an inert or abiotic surface, i.e., mainly characterized by biofilm formation to resist desiccation and starvation.

### Survivability of *S*. Thompson in Leaves With Lesions

In this study, we observed the formation of distinctive macroscopic cell death lesions upon direct inoculation of *S*. Thompson on some basil leaves within 24 h (Figure 3A). In such samples, ST 688C survived slightly better than ST 889B at 1 dpi initially (6.99 ± 0.13 vs. 6.29 ± 0.15 log CFU/sample, *p* < 0.05) but this difference in survivability was no longer present at 6 dpi (*p* > 0.05, Figure 3B). More notably, for both *Salmonella* strains, the bacterial counts were drastically higher in the leaf samples with lesions than the healthy-looking leaf samples at 6 dpi (ST 688C: 4.39 ± 0.68 vs. 2.18 ± 0.29; ST 889B: 4.78 ± 0.12 vs. 2.83 ± 0.18 log CFU/sample, *p* < 0.01, Figures 2B, 3B). The frequency at which the lesions formed on pre-harvest basil leaves was significantly lower (*p* < 0.05) for the clinical-isolate ST 688C at 36.5% (35/96 tested leaves across 10 basil plants) as compared to that of the basil-isolate ST 889B at 59.8% (70/117 tested leaves across 12 basil plants; Figure 3C). In other words, the basil-isolate ST 889B could induce the formation of these cell death lesions in basil leaves significantly better than the clinical counterpart ST 688C. Our fluorescence confocal microscopy of the lesions confirmed that although *Salmonella* grew luxuriantly, they were confined within the lesions while the healthy tissue was devoid of *Salmonella* cells (Figure 3A).

Plants, lacking the highly specific acquired immune systems of higher animals, feature a generalized multilayer immune system designed to resist invasion from a large variety of potential pathogens. The first layer of the plant immune system is the recognition of pathogen-associated molecular patterns (PAMPs), after which the plant mounts an immune response called the PAMP-triggered immunity (PTI; Monaghan and Zipfel, 2012; Garcia and Hirt, 2014). In response, pathogens well-adapted to the host plant can use bacterial delivery systems such as the type III secretion system (T3SS) to suppress the host PTI response (Garcia and Hirt, 2014). Upon failure of PTI, plants typically resort to the second layer of their immune system called the effector-triggered immunity (ETI). This typically leads to the hypersensitive response (HR), characterized by localized cell death lesions, designed to restrict the spread and growth of pathogens (Morel and Dangl, 1997; Heath, 2000a,b; Heidrich et al., 2012).

We next dedicated our efforts to shedding light on the possible underlying basis of ST 889B’s ability to induce the formation of lesions in basil leaves more successfully. Since PTI precedes ETI, which leads to HR, a successful evasion or suppression of PTI must occur for the subsequent induction of HR. It has been reported that in *Salmonella*, flg22 epitome at the N-terminal region of bacterial flagellin is the most well-characterized PAMP (Gómez-Gómez and Boller, 2000; Zipfel et al., 2004) that leads to PTI in plants. Hence, we investigated if any downregulation of the flagellin gene *fliC* by ST 889B to mask its presence and evade PTI was at work. No significant difference was identified in the fold change in the expression level of the *fliC* gene between the two strains (Figure 3D) upon inoculation onto the basil leaves. Hence, the difference in *fliC* gene expression levels between the two strains was promptly eliminated as a possible basis for their differential ability to induce HR in basil leaves.

Subsequently, we explored the possible involvement of the T3SS in the formation of these cell death lesions. Although the role of T3SS and its effectors of a plant pathogen like *Pseudomonas syringae* is very well established (Jones and Dangl, 2006) that of the *Salmonella* T3SS in colonization, proliferation and eliciting the HR in plants is, unfortunately, less unambiguous. The *spaS*^−^ and *sipB*^−^ T3SS mutants showed increased colonization in the alfalfa roots and wheat seedlings (Iniguez et al., 2005) while the *prgH*^−^ and *ssaV*^−^ T3SS mutant counts decreased in *Arabidopsis thaliana* (Schikora et al., 2011). Chalupowicz et al. (2018), on the other hand, observed no significant difference in survivability of wild type *S*. Typhimurium ATCC 14028 to those of various T3SS mutants (*invA*^−^, *ssaV*^−^, *sifA*^−^, and *sipB*^−^) in lettuce leaves.

More pertinently, the wild type *S*. Typhimurium ATCC 14028 also failed to elicit HR in beetroot tissue and pepper leaves when directly inoculated. However, its T3SS effectors SipB and SifA successfully elicited HR when they were delivered instead by plant pathogens *Erwinia amylovora* and *Xanthomonas euvesicatoria*, suggesting that *Salmonella* T3SS effectors, if successfully translocated into plant cells, can cause HR, but the *Salmonella* T3SS machinery is incapable of doing so (Chalupowicz et al., 2018). In contrast, we observed the development of HR cell death lesions within 24 h by direct inoculation of *S*. Thompson onto the basil leaves. This may be due to the high initial inoculum (10^8^ CFU per leaf sample) used in this study. The T3SS has been shown to be an important factor for virulence at high (~10^8^ CFU per plant) but not low titers of *Salmonella* in tomato plants (De Moraes et al., 2017) and *Arabidopsis* (Schikora et al., 2011), although the underlying reason was unclear. High bacterial inoculum may have allowed *Salmonella* to secrete T3SS effectors at levels sufficient to suppress PTI and elicit HR from the basil leaf without translocating into the plant cells directly. Alternatively, the exact outcome of the interaction may also be contingent upon the specific plant species, cultivar., and the *Salmonella* serovar in question (Klerks et al., 2007; Barak et al., 2011). Further research involving multiple plant pathosystems with multiple *Salmonella* serovars need to be conducted to shed light on this matter.

Despite prevailing mixed opinions and results, particularly on the *Salmonella* T3SS and the plant HR as outlined above, generally, the role of the T3SS in triggering HR is well established in many plant pathosystems (Jones and Dangl, 2006). Hence, in this study, we compared the gene expression level of *Salmonella* T3SS gene *prgH*, which has been employed as the reporter indicative of the overall T3SS activity in *Salmonella* (Clark et al., 2009; Wang et al., 2017) in the two *S*. Thompson strains immediately upon inoculation to the basil leaves. We observed a significantly higher (*p* < 0.05) 4.33-fold change in the mean expression level of *prgH* in ST 889B than ST 688C (Figure 3D). First, these results allude to possible involvement of the T3SS in suppressing the PTI and subsequently eliciting HR, specifically in the *S*. Thompson and basil plant pathosystem. Second, a higher T3SS activity may be responsible for the enhanced ability of ST 889B over ST 688C to cause HR in basil leaves.

The observed differences in the gene expression levels despite remarkably similar genetic makeup, which ultimately led to the differences in phenotype and survivability on pre-harvest basil leaves, might in part be attributed to the inherently different natures of the two *S*. Thompson strains. It is known that pathogenic bacteria such as *Salmonella* spp. possesses two distinct “lifestyles,” one tuned for virulence and one for environmental persistence, with each its own set of gene expression profiles as comprehensively reviewed elsewhere (Desai and Kenney, 2019). ST688C, being isolated from a human host with clinical salmonellosis, might have its gene expression profile tuned to that of human host virulence, which may partially explain its lower biofilm formation ability. Conversely, ST889B, being isolated from basil, might have its expression profile tuned to that of environmental persistence. However, whether allowing the clinical ST688C strain to be grown and adapted in basil over an extended time will improve its survivability to match that of ST889B is currently unknown and is a subject of further study.

## Conclusion

In this study, we demonstrated that *S*. Thompson was capable of causing cell death lesions congruent with HR elicited from the basil plant. Furthermore, given the remarkable genetic similarity between ST 889B and ST 688C, the observed differences in phenotypes such as biofilm-forming ability, virulence (lesion formation rate), and survivability on pre-harvest basil could be attributed to the variations in expression levels of the same set of genes rather than to the differences in their genetic makeup. We also highlighted the two distinct *Salmonella* survival strategies in the basil pathosystem (biofilm formation on the healthy-looking leaf surface and elicitation of cell death lesions with subsequent invasion). Gene expression polymorphism may be an important strategy in the adaptation by human pathogens to survive and persist in plants, as demonstrated in this *S*. Thompson-basil model.

## Data Availability Statement

The original contributions presented in the study are publicly available. This data can be found at: https://www.ncbi.nlm.nih.gov/search/all/?term=PRJNA697834.

## Author Contributions

YZ and DL designed the experiments and wrote the manuscript. YZ carried out the experiments with contributions from MT, XP, and CW. All authors have read and reviewed the manuscript.

## Funding

This study was supported by an Advanced Manufacturing and Engineering (AME) Young Individual Research Grant (YIRG; A1984c0042) funded by A-STAR, Singapore.

## Conflict of Interest

The authors declare that the research was conducted in the absence of any commercial or financial relationships that could be construed as a potential conflict of interest.

## Publisher’s Note

All claims expressed in this article are solely those of the authors and do not necessarily represent those of their affiliated organizations, or those of the publisher, the editors and the reviewers. Any product that may be evaluated in this article, or claim that may be made by its manufacturer, is not guaranteed or endorsed by the publisher.

## Footnotes

^1^ https://cge.cbs.dtu.dk/services/Assembler/

^2^ https://www.ncbi.nlm.nih.gov/

^3^ http://tree.bio.ed.ac.uk/software/figtree/

^4^ https://www.ncbi.nlm.nih.gov/tools/primer-blast/

## Supplementary Material

Supplementary Figure S1 *aac(6')-Iaa* gene sequences of ST 688C (template) and ST 889B (query).
