# Supplementary figures and images for: Differential Survivability of Two Genetically Similar Salmonella Thompson Strains on Pre-harvest Sweet Basil (Ocimum basilicum) Leaves
Source: Front Microbiol. 2021 Dec 7;12:740983. doi: 10.3389/fmicb.2021.740983 (PMC8689135; doi:10.3389/fmicb.2021.740983)

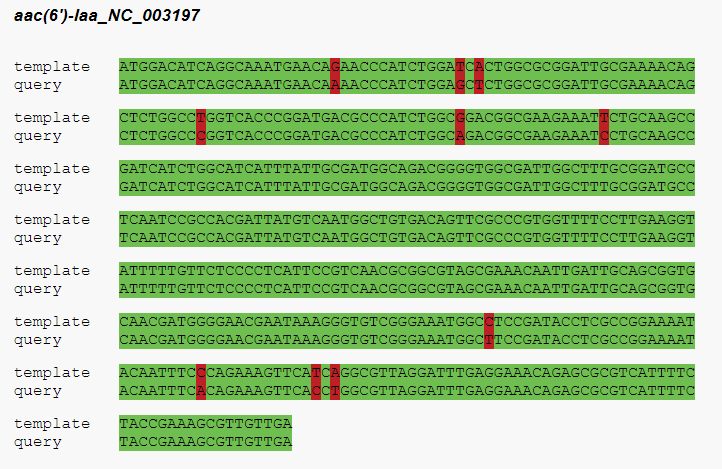

Supplement: Supplementary Figure S1 — aac(6')-Iaa gene sequences of ST 688C (template) and ST 889B (query). [file Image_1.PNG]
